# Newly graduated registered nurses’ experiences of work-integrated learning, and its association with job satisfaction and stress: A cross-sectional study

**DOI:** 10.1016/j.ijnsa.2026.100673

**Published:** 2026-09-05

**Authors:** Anna Andersson, Eva Brink, Gunne Grankvist, Maria Skyvell Nilsson

**Affiliations:** a Department of Health Sciences, University West, SE-46132, Trollhättan, Sweden; b Department of Social and Behavioural Studies, University West, SE-46132, Trollhättan, Sweden

**Keywords:** Job satisfaction, Learning, Newly graduated registered nurses, Stress, Professional development, Regression analysis

## Abstract

**Background:**

The primary focus of work-integrated learning for newly graduated registered nurses is the ongoing development of professional roles, knowledge, and skills. Little is known about how integrating such learning into daily work could influence newly graduated registered nurses’ experiences of job satisfaction and stress. The *aim* of this study is to explore the associations among newly graduated registered nurses’ experiences of work-integrated learning, job satisfaction, and stress.

**Design:**

This study employed a cross-sectional research design.

**Settings:**

A survey was distributed to a sample of newly graduated registered nurses employed at two rural hospitals and one university hospital in Sweden, across departments such as emergency, medical, and surgical units.

**Participants:**

A total of 221 newly graduated registered nurses within their first two years of practice participated in the study.

**Methods:**

The survey included demographic data, as well as the instruments Experienced Work-Integrated Learning, the Short Index of Job Satisfaction, and the Single-Item Measure of Stress Symptoms. Two multiple regression models were analysed to explore associations between the variables.

**Results:**

The results indicate that the experienced learning dimension ‘Developing a relational nursing role’ is positively related to job satisfaction (β: .18; C.I: -.00, .39; p: .05) and that the experienced learning dimension ‘Striving for confidence in medical-technical performance’ is negatively related to stress (β: -.29; C.I: -.06, .18; p: .03).

**Conclusion:**

Our findings indicate that learning plays a role in increasing job satisfaction and reducing stress among newly graduated registered nurses. It is recommended that healthcare organizations should prioritize work-integrated learning opportunities, which are essential for the development of both relational nursing roles and medical-technical competencies, in both daily clinical practice and emergency situations.

**What is already known**

• New nurses experience stress during the transition to their new professional roles
• *New nurses experience a lack of competence and feelings of unpreparedness*
• *Work-integrated learning supports the development of professional competence*

Alt-text: Unlabelled box dummy alt text

**What this paper adds**

• Work-integrated learning is associated with job satisfaction and stress among new nurses
• Learning the relational nursing role is associated with job satisfaction
• Limited learning in medical-technical performance is associated with stress

Alt-text: Unlabelled box dummy alt text

## 1 Introduction

Retaining nurses within the profession is essential for addressing the increasing and evolving care demands within the healthcare system (World Health Organization, 2025). A particularly vulnerable group is newly graduated registered nurses in transition. While managing daily clinical practice, they are expected to deepen their practical and theoretical knowledge and further establish their professional roles (Detlín et al., 2022). For newly graduated registered nurses, this work-integrated learning is emphasized as a multidimensional learning process that involves developing professional roles and acquiring the knowledge and skills required to effectively address healthcare demands (Andersson et al., 2022).

However, the transition period for newly graduated registered nurses is characterized as demanding, both emotionally and cognitively. They experience a gap between their educational preparation and the realities of clinical practice. These discrepancies may contribute to role confusion and feelings of inadequacy (Duchscher, 2009). Such transition experiences have been shown to negatively influence job satisfaction, stress levels, and retention intentions among newly graduated registered nurses (Baharum et al., 2023). However, other research studies have indicated that higher job satisfaction (Kim and Shin, 2020) and lower stress levels (Reebals et al., 2022) are key factors in facilitating a smooth and successful transition into professional nursing practice. Consequently, this study aimed to explore the association between dimensions of work-integrated learning, job satisfaction, and stress among newly graduated registered nurses.

## 2 Background

Learning from clinical experiences is vital for the development of expertise in nursing, that is, high proficiency in qualitatively handling nursing care in various clinical situations (Benner, 1984). Clinical experiences are especially important for newly graduated registered nurses in translating theoretical educational knowledge into practical care situations (Kaldal et al., 2023). Work-integrated learning is often used in higher education contexts and includes a variety of pedagogical activities aimed at helping students connect theoretical knowledge with practical situations, ultimately strengthening their ability to navigate the demands of working life (Zegwaard et al., 2023). In the present study, work-integrated learning refers to a multidimensional learning process within daily work environments (Björck and Willermark, 2025). This learning process involves newly graduated registered nurses engaging in learning, socializing, and developing collaborative and nursing competencies. Work-integrated learning for newly graduated registered nurses also encompasses the development of knowledge and skills to effectively navigate healthcare procedures, for example, ward-specific nursing and medical responsibilities, as well as gaining confidence in medical-technical performances and an experience-based understanding of clinical practices (Andersson et al., 2022).

Previous research indicates that newly graduated registered nurses constitute a particularly vulnerable group in terms of stress (Feeg et al., 2022; Labrague and McEnroe‐Petitte, 2018). Research studies have described several sources of stress for newly graduated registered nurses. For example, they may experience knowledge deficits and clinical unpreparedness (See et al., 2023), high work expectations and responsibilities, fear of making mistakes (Feeg et al., 2022), a lack of professional nursing competence (Kaldal et al., 2023; Labrague and McEnroe‐Petitte, 2018), and lower task mastery, role clarity, and social acceptance (Frögéli et al., 2019). Heavy workloads have also been described as a main contributor to stress (Feeg et al., 2022; Halpin et al., 2017). Furthermore, an integrative review has indicated that stress is one factor contributing to newly graduated registered nurses’ intention to leave the profession (Lyu et al., 2024). Considering the extent and consequences of stress, focusing on reducing stress for newly graduated registered nurses seems especially important. Reebals et al.(2022) specifically acknowledged the importance of supporting competency development to meet workplace expectations and nursing responsibilities in order to ease newly graduated registered nurses’ stressful and challenging transition experiences.

Job satisfaction refers to an individual’s overall attitude toward their work (Brayfield and Rothe, 1951). Job satisfaction has been demonstrated to be a significant factor in reducing newly graduated registered nurses’ turnover intentions (Bae, 2023; Napolitano et al., 2024) and increasing their intentions to stay in the profession (Yarbrough et al., 2017). Research shows that learning and development opportunities are associated with job satisfaction among newly graduated registered nurses (Napolitano et al., 2024; Ulupinar and Aydogan, 2021). A recent survey study by Aiken et al. (2024) of 6643 general nurses across 64 hospitals in six European countries found that approximately 24% of the nurses reported low job satisfaction, 26% had experienced high burnout, 33% expressed intentions to leave, and 29% reported poor or fair health. The authors highlight the need for organizational interventions to improve hospital work environments and support nurses’ well-being.

Against this background, the aim of the present study was to explore the associations among experienced work-integrated learning, job satisfaction, and stress in a sample of newly graduated registered nurses. The specific aims of this study were: (i) to test the validity of the Swedish version of the *Short Index of Job Satisfaction*; (ii) to identify which experienced work-integrated learning dimensions are associated with job satisfaction; and (iii) to identify which experienced work-integrated learning dimensions are associated with stress.

## 3 Methods

### 3.1 Study design

This study utilized a survey methodology and a cross-sectional design to investigate a sample of newly graduated registered nurses.

### 3.2 Study setting and recruitment

The inclusion criteria for participation comprised newly graduated registered nurses within their first two years of practice who had completed a three-year bachelor’s nursing programme. The study included participants from three major hospitals in western Sweden, comprising two rural hospitals and one university hospital. The newly graduated registered nurses worked in various departments, such as emergency, medical, and surgical units. The three selected hospitals had all instituted a one-year introductory programme for newly graduated registered nurses. Each programme had a co-ordinator responsible for introducing newly graduated registered nurses to the care units, providing them with learning activities, and offering support from preceptors and mentors during the introductory period.

### 3.3 Measures

An online survey was conducted using Microsoft 365 (Forms). The survey began with demographic items, such as age, gender, and months of experience as a newly graduated registered nurse. This was followed by the measurement tools *Experienced Work-Integrated Learning (E-WIL),* the *Short Index of Job Satisfaction (SIJS)*, and the *Single-Item Measure of Stress Symptom*.

#### 3.3.1 Experienced work-integrated learning (E-WIL)

The *Experienced Work-Integrated Learning (E-WIL)* instrument used in this study was developed and validated by Andersson et al. (2023) and comprises seven learning dimensions focused on newly graduated registered nurses’ professional development. The first four learning dimensions evaluate newly graduated registered nurses’ personal mastery of their professional roles: *Developing a self-directed learning role* evaluates newly graduated registered nurses’ engagement and reflection on their own knowledge and learning needs; *Developing a relational nursing role* evaluates newly graduated registered nurses’ use of nursing theories, concepts, and essential nursing approaches in clinical practice situations; and the *Transitioning to a collegial role* and *Transitioning to a collegial learning role* evaluate newly graduated registered nurses’ development of the ability to collaborate with coworkers in care and learning situations. The three remaining dimensions evaluate the learning of knowledge and capabilities linked to the demands of healthcare organizations: *Developing contextual workplace knowledge and understanding* on nursing and medical responsibilities of specific wards; *Striving for confidence in medical-technical performance* in new and emergency situations; and *Developing an experience-based understanding of clinical situations*, that is, the development of clinical insight and the capacity to act proficiently in clinical care situations (Andersson et al., 2022).

The *Experienced Work-Integrated Learning* instrument includes seven subscales (dimensions) comprising 29 items, rated using a five-point Likert scale, from 1, ‘do not agree at all’, to 5, ‘completely agree’, with higher values representing a higher level of *Experienced Work-Integrated Learning*. In a previous study, Cronbach’s alpha for the *Experienced Work-Integrated Learning* scale dimensions ranged from .63 to .81 (Andersson et al., 2023), similar to the findings of the present study.

#### 3.3.2 Short index of job satisfaction

The *Short Index of Job Satisfaction* (SIJS) is a self-reported scale comprising five items. The original 18-item Index of Job Satisfaction (Brayfield and Rothe, 1951) was reduced to five items and validated by Judge et al. (2000). The shorter version was used in this study, with respondents rating the five items on a five-point Likert scale, from 1, ‘do not agree at all’, to 5, ‘completely agree’, with higher values representing a higher level of job satisfaction. Two of the items required reverse coding: ‘Each day at work seems like it will never end’, and ‘I consider my job to be rather unpleasant’. In a previous study, Cronbach’s alpha for the original five-item *Short Index of Job Satisfaction* scale was .92 (Judge et al., 2000); in the present study, it was .82.

#### 3.3.3 Single-item measure of stress symptoms

The *Single-Item Measure of Stress Symptoms* measures an individual’s general experience of stress and was originally developed from an occupational stress questionnaire (Elo et al., 1992) and validated as a single-item assessment (Elo et al., 2003). Stress refers to a situation in which a person feels tense, restless, nervous, or anxious, or is unable to sleep at night because his or her mind is troubled all the time. The stress item was rated using a five-point Likert scale, from 1, ‘not at all’, to 5, ‘very much’.

### 3.4 Data collection

Initially, the hospital’s programme co-ordinators were contacted to help recruit prospective newly graduated registered nurses within their first two years of practice. The first author was invited to participate in their programme seminars, where potential respondents were informed and received individual codes. Data collection was then carried out through an online survey during the COVID-19 pandemic, from October 2021 to March 2022. Due to the pandemic, potential participants were initially approached during their physical participation in seminars and later directly via email. A total of 433 newly graduated registered nurses were approached (190 while attending physical seminars and 243 via email).

### 3.5 Data analysis

All statistical analysis was performed using the software program IBM SPSS Version 28.0. Descriptive statistics (*M* and *SD*) were used to explore the included variables. A principal components analysis was performed using SPSS to evaluate the one-dimensional Swedish version of Judge et al.’s (2000) five-item *Short Index of Job Satisfaction* scale. The rule of eigenvalue greater than one was used, and to capture acceptable item–factor relationships, a factor value of .30 was set as the lower limit. A total-variance-explained value of 60% was set as the lower limit (Brown, 2015). Furthermore, to assess effect size, Cohen’s *d* was used to compare newly graduated registered nurses’ job satisfaction, measured by the *Short Index of Job Satisfaction*, with a Brazilian multiple-occupation sample and a Portuguese multiple-occupation sample (Sinval and Marôco, 2020). A Cohen’s *d* value of .20–.50 would indicate a medium standardized mean difference between the groups (Cohen, 1988).

Internal consistency was assessed using Cronbach’s alpha, with a score of 0.70 considered an acceptable value (Field et al., 2024). The bivariate (two-tailed) correlation between variables was analysed using Pearson’s correlation coefficient (95% confidence interval) at a statistical significance level of *p* = .05 (Lang and Altman, 2015). In addition, the correlations between months of experience and the variables of stress and job satisfaction were explored. Two multiple linear regression models were tested, using the enter approach to combine the seven *Experienced Work-Integrated Learning* predictor variables simultaneously, with the *Short Index of Job Satisfaction* and stress as dependent variables (see Table 5).

Multiple linear regression models were evaluated using the regression coefficients (*b*), associated confidence intervals, and statistical significance (*p)*. In addition, to evaluate how well the model fits the observed data, the explained variance (R^2^) was used (Lang and Altman, 2015). The sample size was sufficient for regression analysis, since there were at least 10 to 15 cases for each predictor. The total amount of internal missing data was relatively small; the overall data coverage was approximately .99. Collinearity diagnostics were calculated; as a rule of thumb, a variance inflation factor (VIF) above 10 indicates the presence of high multicollinearity (Field, 2024).

### 3.6 Ethical considerations

This study received ethical approval from the Swedish Ethical Review Authority on August 19, 2021 (Dnr 2021–03,785). The researchers adhered to the ethical principles of the World Medical Association Declaration of Helsinki (2001). All participants were provided with information regarding the study’s purpose and procedures, as well as their rights, including the voluntary nature of participation and the option to withdraw at any time without providing a reason (Swedish Research Council, 2017). All participants provided written informed consent to participate in the study and for their data to be published. To protect participant confidentiality, all data have been anonymized.

## 4 Results

### 4.1 Descriptive characteristics

A total of 221 newly graduated registered nurses completed the online survey, resulting in a total response rate of 51,04%. Of the 221 newly graduated registered nurses, the majority were female (86%). The average age of participants was approximately 28 years, and participants had an average of five months of post-graduation experience in the field of nursing. The demographic statistics are presented in Table 1.

**Table 1:** Demographic characteristics of the participants (n = 221).

Variable	
Age M (SD)	28.33 (5.71)
Gender n (%)	
Female	190 (86%)
Male	28 (12.7%)
Missing	3 (1.4%)
Months of experience as a nurse	
M (SD)	5.25 (4.52)

M = Mean, SD = Standard deviation.

The *Short Index of Job Satisfaction* scale was validated in the present sample of newly graduated registered nurses, and the five-item version was confirmed. The scale demonstrated satisfactory psychometric properties. The principal component analysis indicated that the first two eigenvalues were 3 and .90, confirming a one-dimensional scale (*M* = 19,44; SD = 3.85). This one-factor solution explains 60% of the variance in the total sample. The factor loading of each item of the *Short Index of Job Satisfaction* is presented in Table 2.

**Table 2:** Factor analysis of short index of job satisfaction (N = 221).

Job Satisfaction	M±SD	Factor Loading
I feel fairly satisfied with my present job.	3.83 ± 1.02	.83
Most days I am enthusiastic about my work.	3.69 ± 1.01	.83
Each day at work seems like it will never end.	3.82 ± 1.04	.62
I find real enjoyment in my work.	3.81 ± 0.96	.83
I consider my job to be rather unpleasant.	4.29 ± 0.97	.71

M = Mean, SD = Standard deviation.

Between the newly graduated registered nurses’ *Short Index of Job Satisfaction* (*M* = 3.89; SD = .76) and the Brazilian sample (*M =* 3.68; SD = .87), the result indicated a Cohen’s *d* = .33. Similarly, between the newly graduated registered nurses’ *Short Index of Job Satisfaction* and the Portuguese sample (*M* = 3.61; SD = .89), the result indicated a Cohen’s *d =* .26*.* These results indicate a small-to-medium standardized mean difference between the groups.

The overall descriptive statistics and Cronbach’s alpha for the variables are presented in Table 3. The independent Experienced Work-Integrated Learning variables ranged from *M* = 3.40 (SD = .99) to *M* = 4.18 (SD = .64). The *Short Index of Job Satisfaction* items ranged from *M* = 3.83 (SD = 1.02) to *M* = 4.29 (SD = .97). The single-item variable for stress was *M* = 3.22 (SD = 1.12).

**Table 3:** Descriptive statistics of investigated variables (N = 221).

Variables (Number of items)	M ± SD	Cronbach’s α
Experienced work-integrated learning (E-WIL) (29 items)	111.80 ± 15.48	.83
*Subtheme: Personal mastery of professional roles*		
Developing a self-directed learning role (6 items)		
Developing a relational nursing role (6 items)	3.49 ± 0.69	.78
Transitioning to a collegial role (3 items)	3.76 ± 0.70	.82
Transitioning to a collegial learning role (3 items)	3.97 ± 0.91	.83
*Subtheme: Adapting to organizational requirements*	4.01 ± 0.79	.69
Developing contextual workplace knowledge and understanding (5 items)		
Striving for confidence in medical-technical performance (2 items)	4.11 ± 0.67	.75
Developing an experience-based understanding of clinical situations (4 items)	3.40 ± 0.99	.63
Short Index of Job Satisfaction (SIJS) (5 items)	4.18 ± 0.64	.70
The single-item measure of stress symptoms (1 item)	19.44 ± 3.85 3.22 ± 1.12	.82

M = Mean, SD = Standard deviation, SIJS = Short Index of Job Satisfaction.

### 4.2 Correlations

The correlation analyses indicate that the independent variables Developing a self-directed learning role, Developing a relational nursing role, Transitioning to a collegial role, Transitioning to a collegial learning role, and Striving for confidence in medical-technical performance are positively associated with job satisfaction. The independent variable Striving for confidence in medical-technical performance was negatively associated with stress (see Table 4). The results also show that stress was negatively correlated with newly graduated registered nurses’ months of experience (r = −.22; Cl = −.34, −.09; p = .001).

**Table 4:** Pearson’s correlations coefficient, confidence intervals and significance between variables.

Variables	Job Satisfaction	Stress
Developing a self-directed learning role	.24⁎⁎ [.11, .36]	-.03 [−.16, .10]
Developing a relational nursing role	.26⁎⁎ [.13, .37]	.05 [−.08, .18]
Transitioning to a collegial role	.22⁎⁎ [.09, .34]	-.03 [−.16, .10]
Transitioning to a collegial learning role	.14* [.01, .27]	-.01 [−.14, .11]
Developing contextual workplace knowledge and understanding	.08 [−.04, .21]	-.03 [−16, .09]
Striving for confidence in medical-technical performance	.17* [.04, .29]	-.22⁎⁎ [−.34, .09]
Developing an experience-based understanding of clinical situations	.11 [−.02, .24]	-.03 [−16, .09]

⁎ *p*-statistically significant < 0.05 (2 tailed).

⁎⁎ *p* -statistically significant < 0.01 (2 tailed) Confidence intervals (95%) reported in brackets.

### 4.3 Two linear regression models

The regression model that examined the associations between Experienced-Work Integrated Learning variables and job satisfaction was found to explain 12% of the variance in job satisfaction. *Developing a relational nursing role* was identified as a positive independent variable associated with job satisfaction. Similarly, the regression model that examined the associations between Experienced-Work Integrated Learning variables and stress was found to explain 7% of the variance in stress. *Striving for confidence in medical-technical performance* was identified as a negative independent variable associated with stress; see Table 5. The variance inflation factor ranged from 1.59 to 2, indicating low multicollinearity between the independent variables.

**Table 5:** Two linear regression models; experienced-work integrated learning’s associations with job satisfaction and stress.

Variables	Job Satisfaction B	β	*P*	Stress B	β	*p*
Developing a self-directed learning role	.17 [−.02, .35]	.15	.08	-.07 [−.34, .21]	-.04	.63
Developing a relational nursing role	.20 [−.00, .39]	.18	.05*	.20 [−.09, .49]	.13	.18
Transitioning to a collegial role	.11 [−.02, .24]	.13	.11	.09 [−.11, .29]	.07	.40
Transitioning to a collegial learning role	.09 [−.07, 24]	.09	.28	.01 [−.23, .24]	.01	.95
Developing contextual workplace knowledge and understanding	-.15 [−.35, .04]	-.13	.12	-.11 [−.40, .18]	-.07	.47
Striving for confidence in medical-technical performance	.06 [−.06, .18]	.08	.33	-.33 [−.51, .14]	-.29	.03*
Developing an experience-based understanding of clinical situations Model summary:	-.14 [−.36, .08] R^2^ = .12 Adjusted R^2^ = .09, *p* = .001	-.12	.21	.11 [−.21, .43] R^2^ = .07 Adjusted R^2^ = .04, *p = .*03	.06	.50

B - unstandardized regression coefficient, β - standardized regression coefficient, Confidence Intervals (95%) reported in brackets. *-*p* = statistically significant, R² – Proportion of variance in the dependent variable explained by the model. Adjusted R² – adjusted for the number of predictors.

## 5 Discussion

The findings of this study indicate that specific dimensions of work-integrated learning may contribute to job satisfaction and reduced stress among newly graduated registered nurses. Specifically, the most significant learning dimensions identified are those focused on the development of relational nursing competencies and the enhancement of medical-technical skills in daily clinical practice and emergency situations. Transitioning into a new professional nursing role is a complex and demanding process (Duchscher, 2009). A scoping review found that providing newly graduated registered nurses with opportunities for professional development and building their confidence are vital to their success in the first year of practice (Baharum et al., 2023). A recent integrative review highlights that new nurses stress their need to continue developing both practical and theoretical knowledge, as well as their clinical skills (Jafarimanesh et al., 2026).

The study results indicate that the experienced work-integrated learning variable *Developing a relational nursing role* is associated with job satisfaction. This dimension refers to the development of providing daily nursing care, that is, developing theories and competencies related to the professional role of a nurse. Other research studies have found similar results, indicating associations between the development of the nursing role and job satisfaction among newly graduated registered nurses. For example, an Australian longitudinal educational intervention study focusing on newly graduated registered nurses’ development of nursing competencies indicated that learning these competencies contributed to job satisfaction (Charette et al., 2023). Additionally, a Chinese interview-based study exploring factors influencing job satisfaction among newly graduated registered nurses working in hospital settings found that job satisfaction was related to competence development in providing nursing care (Wong, 2024). Yet another survey-based study conducted in four hospitals in Turkey, involving 428 newly graduated registered nurses who considered themselves incompetent in their nursing competencies, reported low levels of job satisfaction (Ulupinar and Aydogan, 2021).

While newly graduated registered nurses often report a sense of satisfaction and fulfilment regarding their roles, describing feelings of ‘achieving something’ and ‘making a difference’ in patients’ lives found in a qualitative systematic review by (See et al., 2023), they also face significant challenges when transitioning into clinical practice. However, an umbrella review by Kaldal et al. (2023) has highlighted that these challenges are especially evident in the relational and managerial aspects of nursing care. The authors emphasize the need for structured learning support to enhance the relational aspects of nursing, which are crucial for delivering safe and high-quality patient care.

The results of the present study indicate that the experienced work-integrated learning variable, ‘Striving for confidence in medical-technical performance’, is negatively associated with stress. This work-integrated learning dimension refers to the development of confidence in performing medical-technical tasks in daily work as well as in emergency situations. Research has revealed similar findings, indicating an association between the development of medical-technical competence and stress among newly graduated registered nurses. For example, a focus-group-based study among newly graduated registered nurses working in acute care hospitals in central Sweden found that newly graduated registered nurses in acute care hospitals struggled with medical-technical competence in the sense that they needed continuous competence development and collegial support to fulfil the required nursing responsibilities (Willman et al., 2021). A Polish interview-based study found that newly graduated registered nurses experienced stress due to a lack of competence in handling medical-technical equipment in critical care situations and, to reduce stress, they requested learning opportunities in simulation centers (Serafin et al., 2022). Della Ratta (2016) conducted an interview study in the United States highlighting the uncertainty and stress experienced by newly graduated registered nurses due to their lack of medical-technical skills in emergency situations and reported that they went from uncertainty and stress to self-reliance due to repeated encounters with deteriorated patients during their first year of nursing practice.

The results indicate a low explained variance (R-squared) in the regression model between the experienced work-integrated learning variables and stress; however, the dimension ‘Striving for confidence in medical-technical performance’ demonstrated a statistically significant relationship with stress. This suggests that managing patient safety in complex, acute care situations that require advanced medical-technical competencies may be particularly stressful for newly graduated registered nurses, a finding consistent with results from a national survey of newly graduated nurses in the United States (Feeg et al., 2022).

The Short Index of Job Satisfaction scale with its one-dimensional, five-item structure was supported by the current sample of newly graduated registered nurses (Field, 2024). To evaluate the job satisfaction levels of the Swedish sample of newly graduated registered nurses, the mean value was compared with the mean values of a Brazilian multi-occupational sample and a Portuguese multi-occupational sample (Sinval and Marôco, 2020). The results indicate a slightly higher mean value among the Swedish newly graduated registered nurses, which can be attributed to various reasons. One possible reason for this difference could be that these newly graduated registered nurses attended an introduction programme, and transition by way of a practice programme has been shown to be associated with increased job satisfaction as found in a cross-sectional study conducted in Ireland, Italy, and Croatia (Napolitano et al., 2024).The descriptive analysis in the present study indicates a negative relationship between newly graduated registered nurses’ months of experience and self-assessed stress. Other studies have reported similar results; for example, an integrative review identified limited work experience as a significant factor associated with increased stress levels among newly graduated registered nurses (Labrague and McEnroe-Petitte, 2018).

This study has practical implications for healthcare organizations seeking to support the professional development, health, and retention of newly graduated registered nurses. Learning support to develop the relational aspects of nursing care is important, as it enables new nurses to integrate relational care into everyday practice and may promote job satisfaction among newly graduated nurses, a factor that also contributes to reducing intentions to leave the profession. Such learning can be supported through collegial seminars, simulation training, or reflective learning activities integrated into daily nursing practice or introduction programmes. However, further research is needed to examine the effects of such pedagogical interventions, for example by evaluating how the development of the relational aspects of nursing care influences job satisfaction over time.

In addition, supporting new nurses in building confidence in their medical-technical performance and improving their ability to manage emergency situations may contribute to reduced stress. For example, a qualitative interview study conducted in Sweden found that simulation-based education has been shown to provide an effective means of improving newly graduated registered nurses’ perceived ability to provide care in acute situations (Sterner et al., 2023). Other interventions that may support this learning include support from more experienced nurses in daily clinical practice. However, further research is also required to determine how such educational interventions affect stress levels among newly graduated registered nurses.

## 6 Limitations

This study’s sample predominantly comprised women. It is a distribution that aligns with the broader gender composition of the nursing profession in Sweden, where women account for 87% and men for 13% of the total number of 182,848 nursing professionals (Swedish National Board of Health and Welfare, 2024). The data were collected during the COVID-19 pandemic, a period characterized by changes in healthcare delivery that may have affected the experiences and responses of newly graduated registered nurses. Therefore, the results should be viewed in light of the pandemic's possible influence. Additional research in post-pandemic contexts is necessary to verify and enhance the study's findings.

The cross-sectional design of this study does not lend itself to drawing conclusions about causal effects; therefore, the present results should be interpreted accordingly (Field, 2024). To provide a more in-depth understanding of the findings, it is essential to further explore how experienced work-integrated learning for newly graduated registered nurses develops longitudinally and/or affects job satisfaction and stress. This study identified significant positive zero-order correlations between job satisfaction and the five key variables of experienced work-integrated learning: *Developing a self-directed learning role, Developing a relational nursing role, Transitioning to a collegial role, Transitioning to a collegial learning role,* and *Striving for confidence in medical-technical performance* (see Table 4), but only one of these remained significant in the final regression analyses. The regression models explained a relatively small proportion of the variance, indicating that the results should be interpreted with caution. Future studies should focus on developing and deepening analytical models for how different dimensions of perceived work-integrated learning are associated with professional development, job satisfaction, and stress among newly graduated registered nurses.

## 7 Conclusion

Our findings indicate that work-integrated learning plays a role in increasing job satisfaction and reducing stress among newly graduated registered nurses. It is therefore suggested that healthcare organizations invest in specific learning activities that are essential for the development of both relational nursing roles and medical-technical competence in both daily clinical practice and emergency situations. By investing in work-integrated learning, healthcare organizations may enhance newly graduated registered nurses’ competencies to deliver high-quality care and also support their health and intentions to stay in the profession. Future research should focus on examining the longitudinal effects of work-integrated learning dimensions on job satisfaction and stress levels among newly graduated registered nurses.

## Funding

This research study did not receive any specific grant from funding agencies in the public, commercial, or not-for-profit sectors.

## CRediT authorship contribution statement

**Anna Andersson:** Writing – review & editing, Writing – original draft, Visualization, Validation, Software, Resources, Project administration, Methodology, Investigation, Formal analysis, Data curation, Conceptualization. **Eva Brink:** Writing – review & editing, Writing – original draft, Visualization, Validation, Supervision, Methodology, Investigation, Formal analysis, Conceptualization. **Gunne Grankvist:** Writing – review & editing, Validation, Supervision, Methodology, Investigation, Formal analysis, Conceptualization. **Maria Skyvell Nilsson:** Writing – review & editing, Writing – original draft, Visualization, Validation, Supervision, Methodology, Investigation, Formal analysis, Conceptualization.

## Declaration of competing interest

The authors declare that they have no known competing financial interests or personal relationships that could have appeared to influence the work reported in this paper.
